# Genome Sequences of *Salmonella enterica* subsp. *enterica* Serovar Infantis Strains from Hungary Representing Two Peak Incidence Periods in Three Decades

**DOI:** 10.1128/genomeA.01735-16

**Published:** 2017-03-02

**Authors:** Tímea Wilk, Móni Szabó, Ama Szmolka, János Kiss, Ferenc Olasz, Béla Nagy

**Affiliations:** a Agricultural Biotechnology Institute of the National Agricultural Research and Innovation Centre, Gödöllő, Hungary; b Institute for Veterinary Medical Research, Centre for Agricultural Research, Hungarian Academy of Sciences, Budapest, Hungary

## Abstract

Four strains of *Salmonella enterica* subsp. *enterica* serovar Infantis isolated from humans (1980 to 1982) and broiler chickens (2016) have been sequenced. They represent the early and recent peak incidences of this serovar in Hungary. Genome sequences of these isolates provide comparative data on the evolution and rise of an endemic *S*. Infantis clone in Hungary.

## GENOME ANNOUNCEMENT

*Salmonella enterica* subsp. *enterica* serovar Infantis is an emerging serovar among humans in several countries in and outside Europe. It seems to be endemic and the most prevalent serovar in broiler flocks of several countries (1–3). In Hungary, *S*. Infantis reached its first peak incidence in humans in the early 1980s and the second in broiler chickens in the 2000s. The genome sequences of early and recent isolates of *S*. Infantis from broiler chickens have recently been published (4, 5), and this report is the last part of our *S*. Infantis genome announcement trilogy. Here, we present the draft genomes of two pansensitive strains (SI15023h and SI220h) from humans representing the first peak incidence from 1980 to 1982 (6) and two multiresistant isolates (SI240/16 and SI1070/16) from broiler chickens from 2016 representing the second, ongoing peak incidence period in Hungary.

Libraries of 626- to 729-bp fragments were prepared from the four strains and 2 × 300-bp Illumina paired-end genome sequencing was performed by Enviroinvest Zrt. (Pécs, Hungary) using Illumina’s MiSeq platform. The read numbers were 8.7 million for SI15023h, 19.9 million for SI220h, 6.8 million for SI240/16, and 10.2 million for SI1070/16. The estimated coverages of the whole genomes were 558×, 1,274×, 410×, and 626×, respectively.

The reads were *de novo* assembled using A5-miseq (7), and the genomes were annotated using the RAST annotation server (8). We set the taxon to *Salmonella enterica* and the genetic code to *11* (archaea, bacteria). For SI15023h, SI220h, SI240/16, and SI1070/16, respectively, we obtained the following data: total lengths of the chromosomal contigs were 4,711,376 bp, 4,690,379 bp, 4,925,279 bp, and 4,983,448 bp; annotated genes found were 4,758, 4,716, 5,048, and 4,985; tRNAs found were 159, 181, 159, and 160; rRNAs found were 49, 52, 48, and 49; and G+C contents were 53%, 53%, 51%, and 51%.

Nearly 100% similarity was found in pairwise comparisons (9) of the chromosomal sequences of strains derived from the same hosts, while the similarity between the human and broiler chicken isolates was around 99.97%. The comparison of the six previous (4, 5) and the four newly sequenced *S*. Infantis genomes revealed that the United Kingdom poultry strain 1326/28 (GenBank accession no. LN649235) shows 99.95% and 99.94% similarity to the human and the broiler chicken strains. The pansensitive SI69/94 (GenBank accession no. NZ_JRXB00000000) is 99.99% similar to both the human and broiler chicken isolates. The multiresistant SI54/04 (GenBank accession no. NZ_JRXC00000000) and the three recent broiler chicken isolates SI3337/12 (GenBank accession no. MIJS00000000), SI757/13 (GenBank accession no. MIJT00000000), and SI786/13 (GenBank accession no. MIJR00000000) are 99.97 to 99.98% similar to the human and >99.99% to the broiler chicken strains.

Analysis of the four genomes suggests the presence of plasmids of ~277 and 49 kb in SI240/16 and a plasmid of >267 kb in SI1070/16, and shows that all four genomes contain additional sequences that cannot be aligned to the genome of the earliest Hungarian broiler chicken isolate SI69/94 (4).

### Accession number(s).

The draft genome sequences of strains SI15023h, SI220h, SI240/16, and SI1070/16 have been deposited in GenBank under the accession numbers MRUU00000000, MRUV00000000, MRUW00000000, and MRUX00000000, respectively.
